# Parallel processing of face and house stimuli by V1 and specialized visual areas: a magnetoencephalographic (MEG) study

**DOI:** 10.3389/fnhum.2014.00901

**Published:** 2014-11-07

**Authors:** Yoshihito Shigihara, Semir Zeki

**Affiliations:** Wellcome Laboratory of Neurobiology, University College LondonLondon, UK

**Keywords:** form perception, hierarchical model of form processing, orientation selective cells, parallel model of form processing

## Abstract

We used easily distinguishable stimuli of faces and houses constituted from straight lines, with the aim of learning whether they activate V1 on the one hand, and the specialized areas that are critical for the processing of faces and houses on the other, with similar latencies. Eighteen subjects took part in the experiment, which used magnetoencephalography (MEG) coupled to analytical methods to detect the time course of the earliest responses which these stimuli provoke in these cortical areas. Both categories of stimuli activated V1 and areas of the visual cortex outside it at around 40 ms after stimulus onset, and the amplitude elicited by face stimuli was significantly larger than that elicited by house stimuli. These results suggest that “low-level” and “high-level” features of form stimuli are processed in parallel by V1 and visual areas outside it. Taken together with our previous results on the processing of simple geometric forms (Shgihara and Zeki, 2013; Shigihara and Zeki, 2014), the present ones reinforce the conclusion that parallel processing is an important component in the strategy used by the brain to process and construct forms.

## Introduction

In this study, which is a continuation of our earlier studies on parallel processing in the brain's visual form systems (Shgihara and Zeki, 2013; Shigihara and Zeki, 2014), we set out to learn whether faces and houses activate the primary visual cortex (V1) and areas outside it sequentially or within the same time frame. The simplest way of doing so was to use magnetoencephalography (MEG), which has a superior temporal resolution, and record the earliest cortical responses that result from viewing face and house stimuli constituted from straight lines. The use of straight lines to constitute readily distinguishable stimuli belonging to the two categories was important because it is generally supposed that the cortical processing of all forms, including faces and houses, has its source in the orientation selective (OS) cells of area V1 (Bruce and Young, 1986; Biederman and Kalosai, 1998; Riesenhuber and Poggio, 1999; Haxby et al., 2000). Yet recent results show that simple geometrical forms constituted from straight lines activate V1 and the surrounding prestriate visual areas (areas V2 and V3), which also have heavy concentrations of OS cells (Zeki, 1978; Tootell et al., 1988; Kourtzi et al., 2003; Yacoub et al., 2008; Tong et al., 2012), within the same time frame, suggesting that, in addition to the hierarchical strategy, the cortex may also employ a parallel one for processing not only simple geometric forms but more complex ones such as faces and houses as well.

We chose faces and houses as our “higher” level stimuli partly because they have a well-known temporal signature, resulting in a cortical activation at 170 ms (although that time interval has been revised downwards, see Discussion). This has been used to argue in favor of a hierarchical strategy, in which “low-level” elements are processed and analyzed first, beginning in V1, before being combined to constitute distinct and more complex forms in “higher” areas outside it. We also chose these stimuli because there are cortical areas outside V1 that are critical for processing of faces and houses, especially the occipital face area (OFA) (Peelen and Downing, 2005; Pitcher et al., 2012), the fusiform face area (FFA) (Sergent et al., 1992; Kanwisher et al., 1997; Kanwisher and Yovel, 2006) and the parahippocampal place area (PPA) (Epstein and Kanwisher, 1998), although whether these areas are uniquely specialized for faces or houses has been debated (Haxby et al., 2001). Such specializations made it more plausible to suppose that the parallel inputs from subcortical stations to prestriate cortex would manifest themselves temporally in an early latency response, comparable to that in V1. In this time-based study, we were not however especially concerned with localizing these areas precisely, our sole concern being to learn whether the responses elicited could be traced to V1 and to areas lying outside it. Nor were we particularly concerned with the characteristic N 170 ms temporal signature of the reaction to face and house stimuli. Rather, our concern was solely with the earliest response provoked by these stimuli in striate and prestriate cortex. In this way, we hoped to supplement our earlier results (Shgihara and Zeki, 2013; Shigihara and Zeki, 2014) and learn whether “low-level” features, generally considered to be processed in V1, and “high-level” features, generally thought to be processed in specialized visual areas outside it, are in fact processed in parallel by V1 and the areas lying in cortex outside it.

We also used faces and houses as stimuli because they constitute two very distinct categories of forms. Faces have a very privileged position in visual perception, one not shared by other categories of form such as houses and other man-made objects, which are known to activate specialized areas of the visual brain as well. Whether due to an inherited brain template or a privileged rapid onset plasticity (Gauthier and Nelson, 2001; Zeki and Ishizu, 2013 for a review), newborn infants orient to faces or face-like stimuli within hours after birth (Goren et al., 1975; Johnson et al., 1991). This made it plausible, intuitively at least, to suppose that faces are processed faster than stimuli such as houses, which belong to man-made categories, even when both sets of stimuli are constructed from the same elements (lines) which are also optimal for activating the OS cells of V1. Our approach thus offered the opportunity of learning whether (a) when constructed from lines, the early activity produced by these two categories of stimuli in V1 and the visual areas outside it occurs within the same time frame; (b) there is any difference in latency of activation between so privileged a stimulus as a face and stimuli depicting man-made artifacts such as houses and, most importantly, (c) whether the relationship between “low-level” (oriented lines) and “high-level” (faces and houses) form processing is chronologically hierarchical. This would be so if the early responses obtained from V1 and from cortex outside it differ temporally.

## Materials and methods

### Subjects and study design

Eighteen right-handed healthy adult volunteers (8 female, mean age 28.3 ± 9.2 years) took part in the study. None had a history of neurological or psychiatric disorder; written informed consent was obtained from all and the study, which conforms to the Code of Ethics of the World Medical Association, was approved by the Ethics Committee of University College London.

### Stimuli and task

Stimuli were generated using Cogent 2000 and Cogent Graphics (http://www.vislab.ucl.ac.uk/cogent.php) toolboxes running in MATLAB (MathWorks, Na-tick, MA, USA) and were rear projected on a screen by a projector (RM-MSX21G, Victor Company of Japan, Kanagawa, Japan) which has a resolution of 800 × 600 pixels at 60 Hz. Trigger signals were recorded for the MEG system through an IEEE 1284 connection. The delay between the trigger signal and the projection of stimuli (33 ms) was confirmed using a photodiode on the screen before scanning subjects and was corrected during data processing.

**Figure 1:**
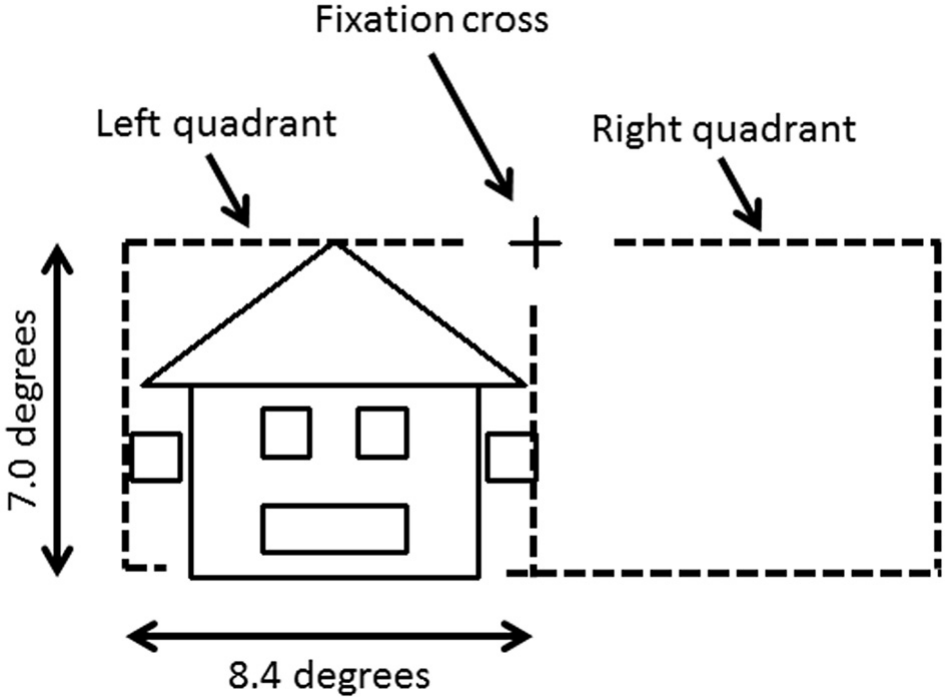
**Stimulus image and projected hemifield**. The fixation cross is located in the center of the screen. Each stimulus was projected in the lower left or right quadrant, and covered up to 7.0° below the fixation cross and up to 8.4° on either side.

**Figure 2:**
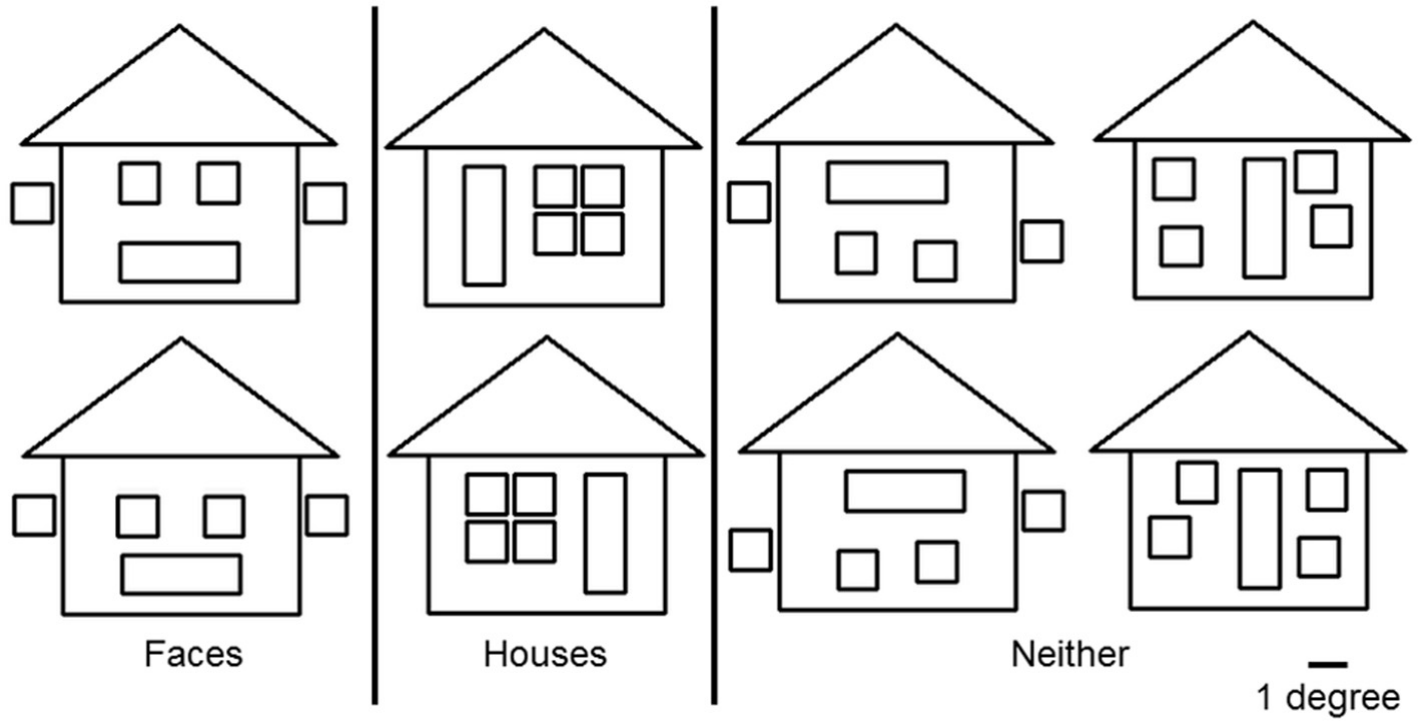
**Three categories of stimuli**. “*Face*” and “*House*” stimuli were composed of the same lines but had configurations that made them easily recognizable as belonging to one category or the other. The “*Neither*” category consisted of four figures that were not easy to categorize as “*Face*” or “*House*.” All figures consisted of the same elements: 6 squares and 1 triangle, of which the maximum heights and width were 7.0° and 8.4° respectively. The width of each line was 0.1°. A white fixation cross (1.0° × 1.0°) was projected at the center of the screen.

To avoid cancelation effects that can occur when both banks of the calcarine sulcus are stimulated (Portin et al., 1999), stimuli were displayed separately in either the lower left or lower right quadrants of the visual field and covered an area of 0° and 7.0° below the fixation cross and 0°–8.4° on either side (Figure 1). Three different categories of stimuli, all composed of the same 26 lines combined into similar forms (six squares and one triangle), were used: faces, houses and ones which could not easily be categorized into either face or house (“*Neither*”). The latter stimuli were used only to ensure that subjects maintained their attention during the experiment. Two different versions of “*House*” and “*Face*” stimuli were used, while the “*Neither*” category had four versions (see Figure 2).

### Scanning details

MEG data were recorded continuously using a 275-channel CTF Omega whole-head gradiometer (VSM MedTech, British Columbia, Canada). Data were sampled at 1200 Hz with a 300 Hz low-pass filter without using high-pass filters. Subjects were fitted with localizer coils at the nasion and at 1 cm anterior to the left and right traguses to monitor head movements during the recording sessions and co-register them to individual MRI structural images acquired in a 3T MRI scanner (Siemens Magnetron Allegra MRI scanner or Trio Tim 3T scanner, Siemens, Erlangen, Germany). Gaze position and blinking were monitored by an EyeLink 1000 eyetracker (SR Research Ltd., Ontario, Canada).

Subjects viewed the display screen at a distance of 60 cm. The experiment consisted of five 5 min runs; each comprised 140 stimuli, 63 of which were “*Faces*,” 63 “*Houses*” while 14 belonged to the “*Neither*” category. The order of stimulus presentation according to category, sub-type and hemifield stimulated (left or right) was randomized. Stimulus presentation lasted 283 ms, with a randomly varied inter-stimulus interval (ISI) of 1500–2000 ms. Subjects focused on the fixation cross throughout the MEG scan and were asked, during the ISI, to indicate which category of stimulus had been presented, by pressing one of 3 buttons using their index, middle, and ring fingers; the ring finger was always used for the “*Neither*” category, whilst the index and middle fingers were counter-balanced across subjects.

### Data processing

Data were analyzed offline using SPM-8 (Wellcome Trust Centre for Neuroimaging, London, UK; http://www.fil.ion.ucl.ac.uk/spm). They were divided into 1000 ms epochs, each starting 517 ms before stimulus onset. Epochs affected by blink artifacts (detected using the eye-tracker and also by manual inspection of the raw signal data) were discarded and the remaining ones averaged in each condition and baseline corrected. About 140 responses were recorded for each subject, category, and quadrant, except for the “*Neither*” category (*Face* and *House* stimuli presented in left quadrant, 139.7 ± 14.8 ms and 141.0 ± 18.4 ms, respectively; *Face* and *House* stimuli presented in right quadrant, 148.2 ± 34.9 ms and 145.1 ± 16.3 ms, respectively; *Neither* presented in left and right quadrants 35.2 ± 7.6 and 30.4 ± 5.4, respectively). MEG data for the “*Neither*” category was ignored in the subsequent analyses due to lack of usable epochs. The signal during the 100 ms period preceding stimulus onset was used as a baseline. Software filters produce artifacts (Acunzo et al., 2012; Ramkumar et al., 2013) and are not recommended (VanRullen, 2011); like others before us (Noguchi et al., 2004; Inui et al., 2006; Acunzo et al., 2012), we therefore analyzed our results without filters.

### Sensor-level analysis

We hypothesized that (1) there is an early component of event-related magnetic fields (ERFs), before 50 ms post-stimulus (ffytche et al., 1995; Inui et al., 2006; Shgihara and Zeki, 2013), and that (2) there would be differences in the amplitude of magnetic responses between *Face* and *House* stimuli at the early component of ERFs. To test these hypotheses, we defined a sensor of interest (SOI) which showed the largest root-mean-square (RMS) amplitude of ERF between 25 and 50 ms among the 37 occipital sensors selected by us based on the sensor names MLO 11–53 and MRO 11–53, defined by SPM-8 (SOI approach: Liu et al., 2002; Noguchi et al., 2004) since our previous work had shown an early component of ERF around this time window (27–44 ms) (Shgihara and Zeki, 2013). SOIs were defined for each subject and for each of the 4 conditions [2 forms (*Face/House*) × 2 quadrants (Left/Right)] separately; the locations of SOIs are shown in the Supplementary Data. We confirmed that there was no difference in the peak time across the four conditions using a Two-Way ANOVA (2 forms × 2 quadrants) with repeated measures (Main effect of form, *P* = 0.619; Main effect of quadrant, *P* = 0.107; Interaction, *P* = 0.690).

**Figure 3:**
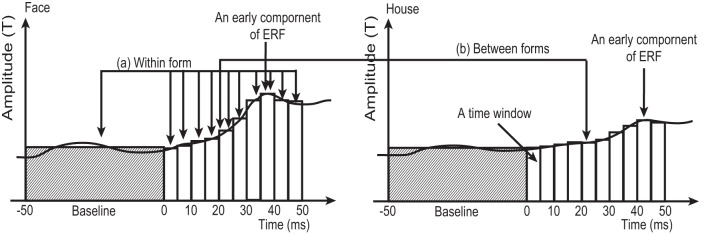
**Schematic figure, not using real data, to illustrate the two types of comparison in the sensor-level analysis employed in this study. (A)** Within form comparison: Average RMS amplitudes at each time window (5 ms in width) between 0 and 50 ms after stimulus onset were compared against the average amplitude during baseline (−50 to 0 ms), using One-Way ANOVA for repeated measures followed by *post-hoc t*-test. This comparison was carried out for 4 conditions (2 forms × 2 quadrants) separately. **(B)** Between forms comparison: Averaged RMS amplitudes at each time window were compared between *Face* and *House* conditions for each quadrant (left/right) separately. White and shaded columns show the averaged RMS amplitude at each time window and baseline, respectively.

**Figure 4:**
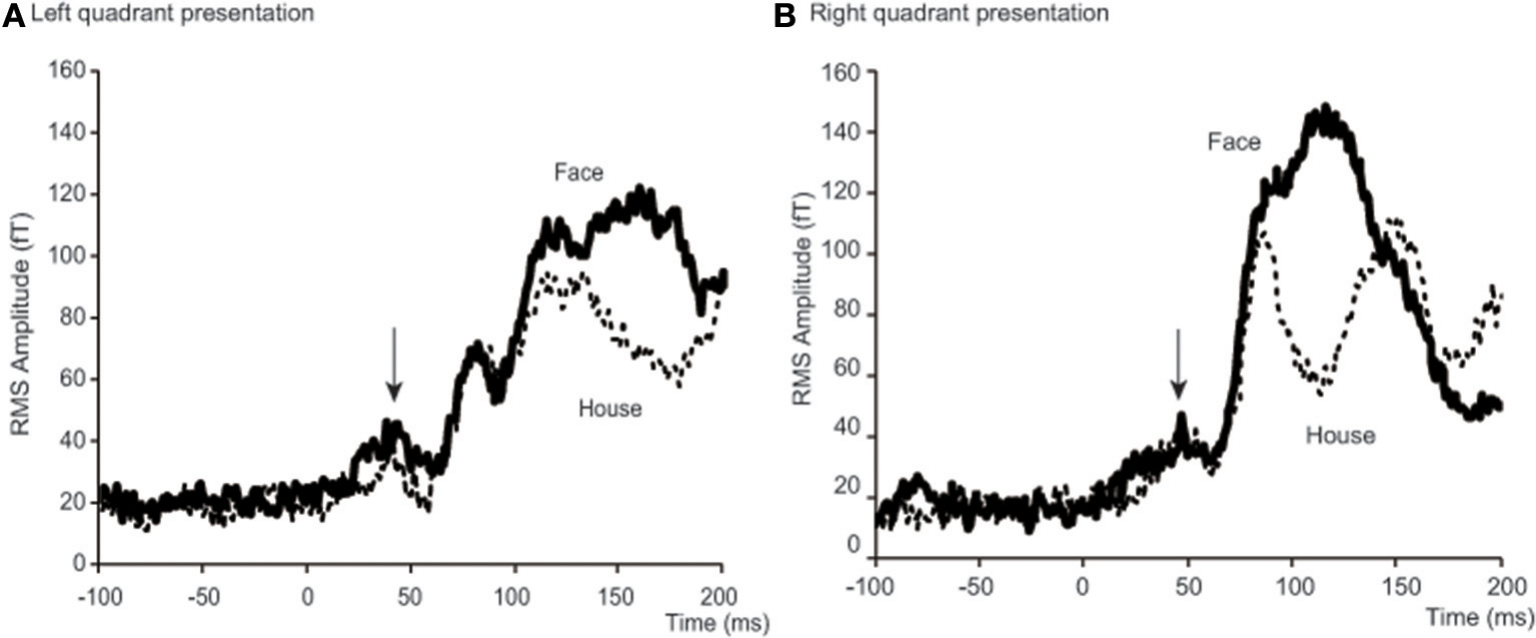
**Time courses of averaged RMS amplitude across 18 subjects**. Each condition shows a peak at around 40 ms after stimulus onset. For left quadrant presentation **(A)**, RMS of amplitude for *Face* stimuli (bold lines) was larger than for *House* stimuli (broken lines) not only after 100 ms, but also before 50 ms after stimulus onset. For right quadrant presentation **(B)**, the difference is only clear after 100 ms. Arrows indicate a peak at around 40 ms after stimulus onset. Although N170 responses are shown between 100 and 200 ms for left quadrant presentation **(A)** and 80 and 150 ms for right presentation **(B)**, we do not discuss these components in this paper, because our interest was with the early responses alone.

Averaged RMS amplitudes at SOIs across 18 subjects showed peaks around 40 ms (37.2–47.2 ms) for all 4 conditions (**Figure 4**). To confirm that these peaks were significantly larger than the baseline level, we performed an analysis which is schematically represented in Figure 3 (within form comparison): We divided our data into ten 5 ms time windows, covering the period between 0 and 50 ms after stimulus onset. This included the early response period described in previous studies (Shgihara and Zeki, 2013), namely 27–44 ms. We also defined a baseline period between −50 and 0 ms before stimulus onset.

Averaged RMS amplitudes at each time window were compared against baseline. This comparison was carried out for 4 conditions (2 forms × 2 quadrants) separately, using One-Way ANOVA with repeated measures followed by *post-hoc t*-tests with Ryan correction (Ludbrook, 1991).

To address the second hypothesis (that *Face* and *House* stimuli produce responses with different amplitudes), RMS amplitudes at each time window were compared between *Face* and *House* stimuli using a paired *t*-test for each quadrant (left/right) separately (Figure 3: Between forms comparison).

### Source-level analysis

Sensor-level analysis revealed that all four conditions produced early components of ERFs at around 40 ms (37.2–47.2 ms) after stimulus onset, and that *Face* stimuli produced larger responses than *House* stimuli for left quadrant stimulation. To learn which brain areas were responsible for producing these responses, we applied source level analyses for the early component of ERFs using SPM-8. Forward modeling was performed between 0 and 50 ms after stimulus onset using a single sphere model (fine mode), and source inversion (estimation) was performed for the peak time window for each condition (shown in Figure 4) using Multiple Sparse Priors (MSP, Greedy Search; Mattout et al., 2005; Friston et al., 2008) for each subject and condition (first level analysis). The source images produced by the first level analysis were smoothed using a Gaussian smoothing kernel of 9 × 9 × 9 mm and taken to the second (between subject) level analysis using *t*-tests within occipital areas defined by WFU_PickAtlas (http://www.nitrc.org/projects/wfu_pickatlas/). The existence of these ERFs was independently established at a statistically significant level for the sensor-level analysis (Shgihara and Zeki, 2013). Here, we report the source locations of peak level activations at a significance threshold of *P*(uncorrected) < 0.001 as well as *P*(FWE corrected) < 0.05. The same source localizations were performed for the other time bins as well. **Figure 6** shows source locations at each time bin and for each condition. These sources were accumulated across time bins for each condition using Image Calculator in SPM-8 to locate the areas which were activated at any time during the early component of the response (**Figure 7**).

## Results

### Behavioral results

Accuracies and response times for subjects' answers were measured during MEG scans (Table 1) and a Two-Way repeated measures ANOVA (quadrant × form) was applied to each analysis. No significant main effect or interaction was found for accuracy [Main effect of Quadrant, *F*_(1, 17)_ = 1.376, *P* = 0.257; Form, *F*_(1, 17)_ = 0.014, *P* = 0.906; Interaction, *F*_(1, 17)_ = 4.178, *P* = 0.057]. Accuracy for each stimulus condition was more than 95%, which shows that the two types of stimuli constituted from the same lines were perceptually accurately, and readily, distinguishable. There were no significant differences between conditions. Although both the main effect of quadrant stimulated and the interaction were not significant, a significant main effect of form was found for response time [Quadrant, *F*_(1, 17)_ = 1.533, *P* = 0.233; Main effect of Form, *F*_(1, 17)_ = 8.057, *P* = 0.011; Interaction, *F*_(1, 17)_ = 0.094, *P* = 0.762]. Differences in form (*Face* and *House*) led to significant differences in terms of response time, which was 20 ms longer for *House* stimuli than *Face* stimuli.

**Table 1:** **Accuracy and response time for button pushing**.

		**Left Average ± *SD***	**Right Average ± *SD***
Accuracy	Face	97.6 ± 3.3%	96.3 ± 6.1%
	House	96.8 ± 5.1%	97.0 ± 5.3%
Response time	Face	667.7 ± 77.8 ms	672.4 ± 88.9 ms
	House	690.7 ± 111.6 ms	698.8 ± 109.7 ms

### Results of sensor-level analysis

**Figure 5:**
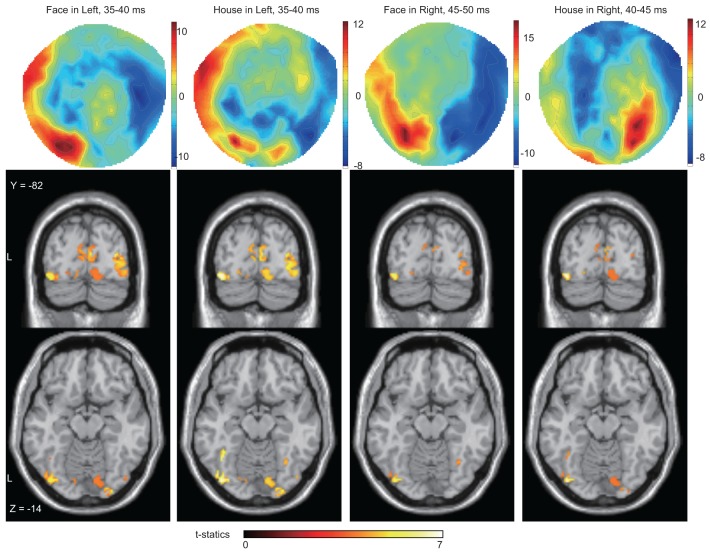
**Averaged contour maps across 18 subjects and statistical parametric maps of estimated source locations of ERFs for group-level analysis (between subjects), superimposed on a standard brain image, broken down by stimulus form and display quadrant**. Source estimation was performed using MSP during peak time window. Contour maps: Red areas represent outflow of magnetic fields and blue areas their inflow, hence the proximity of the two signifies the presence of an electrical current between them. In these areas, at least one part of the red and the blue zones can be traced to occipital sensors; hence each contour map suggests the existence of magnetic sources at least in occipital areas. Statistical parametric maps: Sources were estimated not only around the mid line (striate cortex) but also in other areas for all conditions. The display threshold is at peak level *P*(unc.) < 0.001.

To better define the early component of ERFs, we calculated a RMS of amplitudes at the SOI which showed the largest amplitude among all 37 occipital sensors, between 25 and 50 ms after stimulus onset. The averaged time course of the RMS amplitudes at the SOIs for the four conditions [2 forms (*Face/House*) × 2 quadrants (Left/Right)] and across 18 subjects is shown in Figure 4 and a representative RMS time course for a single subject (Subject #12) is shown in Figure S2 in the Supplementary Data. There is, on average, a peak at around 40 ms after stimulus onset for each condition: 37.2 and 39.7 ms for *Face* and *House* stimuli presented in the left quadrant, respectively; 47.2 and 44.7 ms for *Face* and *House* stimuli presented in the right quadrant, respectively (See Table 2), and the average peak for *Face* stimuli lasted longer than that for *House* stimuli for left quadrant presentation. Contour maps at the peak time bins are shown in Figure 5. They show the existence of magnetic sources in occipital areas for all four conditions, although the activation may extend beyond anteriorly.

**Table 2:** **Results of ANOVA, *post-hoc t*-test, paired *t*-test between two forms (Face vs. House), and Peak time of ERF**.

**Comparison**	**Stimulation**		**ANOVA**				***Post-hoc t*-test**											**Peak (ms)**
							**Time window (ms)**											
			***df***	***F***	***P***		**Baseline**	**0–5**	**5–10**	**10–15**	**15–20**	**20–25**	**25–30**	**30–35**	**35–40**	**40–45**	**45–50**	
Within form	Left	Face	10	5.377	<0.001	t		NS	NS	NS	NS	2.227	3.192	3.505	3.829	4.683	3.289	37.2
						p						0.027	0.002	0.001	<0.001	<0.001	0.001	
		House	10	3.682	<0.001	t		NS	NS	NS	NS	NS	NS	2.237	4.090	3.173	NS	39.7
						p								0.027	<0.001	0.002		
	Right	Face	10	6.403	<0.001	t		NS	NS	NS	NS	2.988	3.047	3.263	3.407	3.588	4.925	47.2
						p						0.003	0.003	0.001	0.001	<0.001	<0.001	
		House	10	6.403	<0.001	t		NS	NS	NS	NS	NS	NS	2.478	3.508	4.716	4.814	44.7
						p								0.014	0.001	<0.001	<0.001	
Between forms	Left	Face vs. House				t	NS	NS	NS	NS	NS	NS	2.329	NS	NS	NS	NS	
						p							0.032					
	Right	Face vs. House				t	NS	NS	NS	NS	NS	NS	NS	NS	NS	NS	NS	
						p												

*Ryan correction was applied for post-hoc t-tests. NS, not significant (p = 0.05). Full description of this table is shown in Table S1 in Supplementary Data*.

To confirm these observations, we performed two analyses on the data, divided into 5 ms time windows, as shown in Figure 3: (a) One-Way ANOVA with repeated measures for each of the four conditions (2 forms × 2 quadrants) to confirm that the peak was larger than baseline (within form comparison in Figure 3) and (b) paired *t*-tests to compare the RMS amplitudes produced by *Face* and *House* stimuli at each of the 10 time windows for each quadrant separately (between form comparison in Figure 3) with the following results:

- A One-Way ANOVA showed a significant main effect of time window for all four conditions (Table 2). *Post-hoc t*-tests showed that the RMS amplitude was significantly larger than baseline at 20–50 ms for *Face* and at 30–45 ms for *House* stimuli presented in the left quadrant, respectively, and at 20–50 ms for *Face* stimuli and 30–50 ms for *House* stimuli presented in the right quadrant, respectively in the right quadrant (Table 2).
- A paired *t*-test showed that the RMS amplitude at 25–30 ms post-stimulus onset was significantly larger in response to *Face* than to *House* stimuli for left quadrant presentation (Table 2, Table S1 in Supplementary Data). This time bin (25–30 ms) precedes the time bin of the peaks (35–40 ms) produced by both stimuli, a finding that matches our observation that the response elicited by *Face* stimuli lasts longer than that elicited by *House* stimuli when presented in the left quadrant. No other differences were found.

In summary, all four conditions showed an early component of ERF at around 40 ms after stimulus onset and ERF amplitudes elicited by *Face* stimuli were different (and larger) than those elicited by *House* stimuli.

### Results for source-level analysis

**Figure 6:**
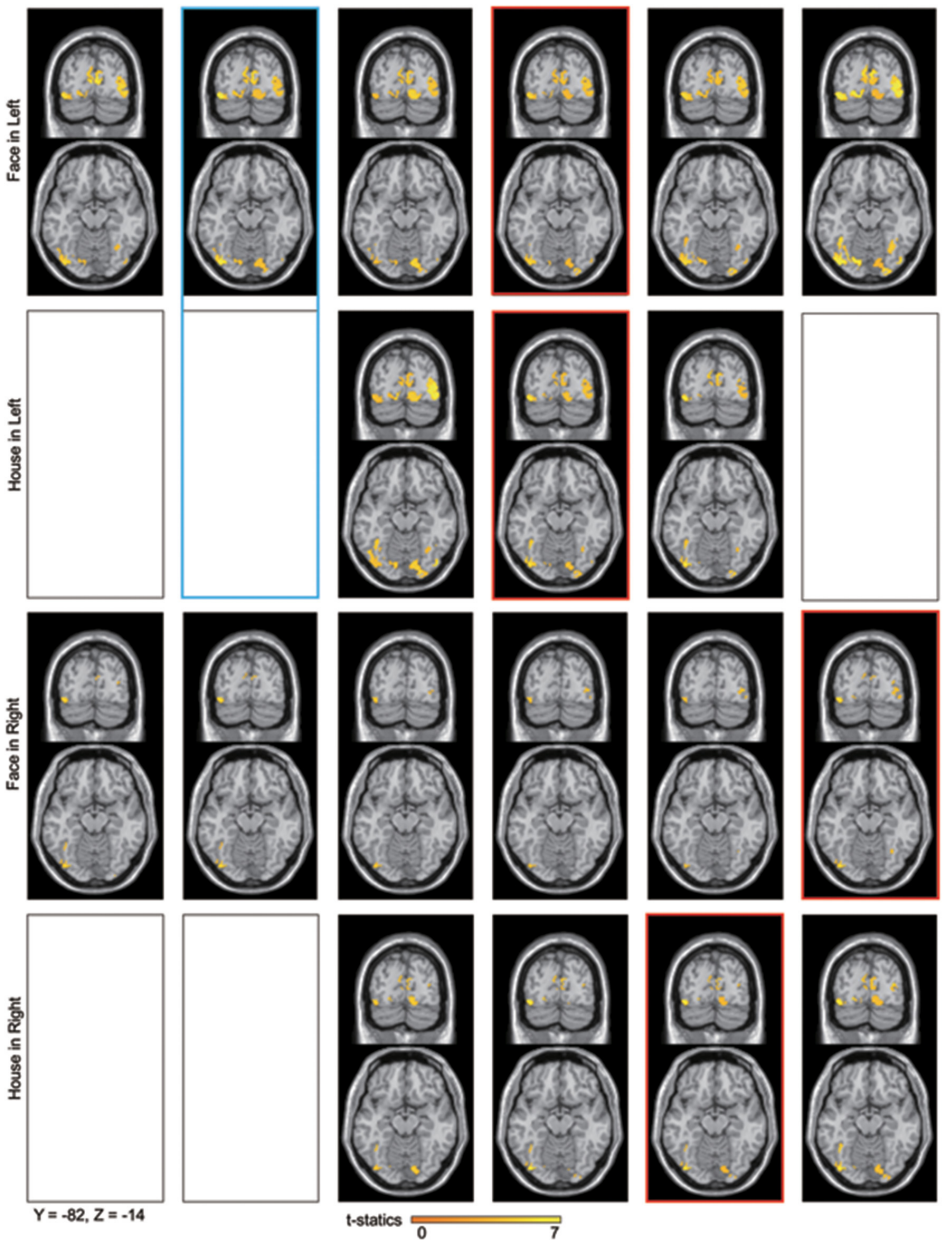
**Averaged contour maps across 18 subjects and statistical parametric maps of the estimated source locations of ERFs for group-level analysis along time bins, superimposed on a standard brain image and broken down by stimulus form and display quadrant**. Source estimation was performed using MSP during time bins and significant activation confirmed by sensor-level analysis. The display threshold is at peak level *P*(unc.) < 0.001. Blue square, a set of conditions which showed significant difference in amplitudes using sensor-level analysis; Red square, peak time bin; Black square, time bin in which significant response was not confirmed by sensor-level analysis.

**Figure 7:**
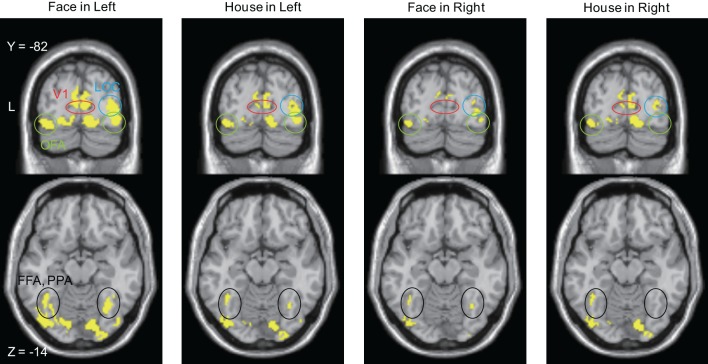
**Brain areas in which sources were estimated at time bins between 20 and 50 ms after stimulus onset**. The display threshold is at peak level *P*(unc.) < 0.001. Red circle, V1 (striate cortex); Green circle, Occipital Face Area (OFA); Blue circle, lateral occipital complex (LOC); Black circle, Fusiform Face Area (FFA), and Parahippocampal Place Area (PPA).

We next wanted to determine the cortical source of the regions producing these ERFs, restricting ourselves to determining whether the source was in V1 as well as the (prestriate) areas outside it, without trying to localize the precise source to previously demarcated regions of the prestriate cortex, a difficult task because of the relatively poor spatial resolution of MEG. Source localization was performed using MSP to estimate the sources of the ERFs during the peak time windows (which corresponds in Figure 3, left panel, to the 35–40 ms bin). See also Table 2 and Figure 5). Figures 6, 7 show the sources at different time windows). At the peak time windows, sources were estimated in both striate (V1) and prestriate cortices for all conditions (Figure 5; Table 3 and Table S2); sources in both were significant at peak level *P* < 0.05 (FWE corrected) for *Face* and *House* stimuli presented in the left quadrant. For right quadrant *Face* stimuli presentation, however, only sources in prestriate cortex were significant while for right quadrant *House* presentation, only sources in striate cortex were significant.

**Table 3:** **Sources locations which were responsible for producing the early components of ERFs**.

**Stimulation**		**Time window (ms)**	**Cluster**			**Peak**			**Coordinate**			**Brain reigion**
			***p*(FWE)**	***kE***	***p*(unc)**	***p*(FWE)**	***T***	***P*(unc)**	***X***	***Y***	***Z***	
Left	Face	35–40	0.015	376	0.026	0.008	6.27	<0.001	36	−80	6	Prestriate
			0.070	159	0.128	0.030	5.42	<0.001	−40	−82	−12	Prestriate
			0.005	555	0.009	0.038	5.28	<0.001	6	−80	14	Striate
						0.040	5.24	<0.001	−6	−78	18	Striate
			0.047	210	0.084	0.040	5.25	<0.001	26	−94	−18	Prestriate
	House	35–40	0.088	158	0.249	0.005	6.29	<0.001	−40	−74	−10	Prestriate
			0.211	29	0.638	0.020	5.40	<0.001	−38	−50	−14	Prestriate
			0.036	339	0.099	0.039	4.99	<0.001	36	−80	6	Prestriate
			0.026	418	0.070	0.040	4.98	<0.001	6	−80	14	Striate
Right	Face	45–50	0.127	94	0.263	0.011	5.98	<0.001	−40	−82	−12	Prestriate
			0.323	9	0.756	0.049	5.06	<0.001	34	−62	−16	Prestriate
	House	40–45	0.128	89	0.225	0.003	6.87	<0.001	−40	−82	−12	Striate

*Statistical values, Cluster size, Location, MNI co-ordinates are displayed. In this table, all sources are peak level significant at p < 0.05 (FWE corrected). Sources locations with peak level significant at p < 0.001 (uncorrected) is shown in Table S2 in Supplementary Data*.

Sources for other time windows are shown in Figures 6, 7. All four conditions show sources in prestriate cortex which apparently include areas V3, OFA, FFA, PPA, and lateral occipital complex (LOC), although these source locations are not as accurate as one would wish, due to technical limitations (see Discussion).

Sensor-level analysis showed a difference in amplitudes between *Face* and *House* stimuli for left quadrant presentation at 25–30 ms after stimulus onset. Although *House* stimulation did not produce a significant response against the baseline, *Face* stimuli did so and the sources of the response were localized in both striate and prestriate cortex (Figure 6, Blue square). This shows that the difference in amplitude at the earliest time window is responsible for both striate and prestriate cortex responses.

In summary, *Face* and *House* stimuli presented in the left quadrant activated both V1 (striate cortex) and the prestriate cortex outside it with a peak at around 40 ms after stimulus onset, with the activation produced by *Face* stimuli being larger in amplitude than those produced by *House* stimuli. For stimuli presented in the right quadrant, activities were less evident at around 40 ms after stimulus onset, in both striate and prestriate cortex. During the whole period of the early component of ERFs, both striate and prestriate cortex were activated for all four conditions in both hemispheres.

## Discussion

Our results show, in summary, that (a) *Face* and *House* stimuli constituted from straight lines produce an early response around 40 ms post-stimulus; (b) the amplitude of the response produced by *Face* stimuli is significantly larger than that produced by *House* stimuli when presented in the left quadrant; and (c) both sets of stimuli produce early activity in both striate and prestriate cortex when presented in the left quadrant but that activity produced by them following right quadrant stimulation was less easily localizable because the statistical power for locating them was less significant.

The view that, in the cerebral cortex, the processing of visual forms (of which faces and houses constitute two examples) begins in V1, coupled to the well documented late latency (N170) in response to both categories of stimuli (Bentin et al., 1996), has fortified the belief that a hierarchical strategy, whose source is in the OS cells of V1, is the ubiquitous one used for the processing of forms, including faces. Our present results, together with our previous ones (Shgihara and Zeki, 2013; Shigihara and Zeki, 2014), suggest however that a parallel strategy, involving both V1 and specialized visual areas outside it, may also be used to process forms, in addition to the hierarchical strategy.

### Hierarchical and parallel processing and the temporal order of activation of visual areas

The temporal signature, at which a robust response is elicited from cortex in response to face and house stimulation is the N170, which is in the 130–170 time window post-stimulation (Rousselet and Husk, 2008). This means that activity in response to face or house stimulation cannot be distinguished earlier than this time frame, the time up to that period being considered to be taken up by processing of “low-level” features of form (e.g., those of orientation, spatial frequency and contrast, see Johnson and Olshausen (2003) and Rousselet and Husk (2008). 170 ms is considerably longer than the latency with which a response is obtained from V1 and longer than the latencies of the main component of visual perception (i.e., N75, P100, and N145) (see review by Tobimatsu and Celesia, 2006). The difference in latency between the early (N75) response to visual stimulation and the late (N170) component corresponding to the recognition of stimuli as distinct is consistent with a hierarchical strategy in which “low-level” features are processed first. More recently, the latency of activation produced by face stimuli, at least, has been revised downwards. In particular Seeck et al. (1997), using intra- and extra-cranial electrodes, obtained activity at 50 ms post-stimulus when subjects were asked to differentiate between familiar and unfamiliar faces. Other studies using MEG have also shown that face stimuli lead to activation in occipito-temporal cortex before 100 ms (Braeutigam et al., 2001; Meeren et al., 2008) As well, transcranial magnetic stimulation studies (Pitcher et al., 2007) have shown that face perception can be disrupted at 40–50 ms post-stimulus. Although these results might suggest that the cortical processing of faces (and abstract objects) occurs much earlier than previously thought, these evoked response studies used photographs of actual faces as stimuli to elicit responses, which raises the question of whether the early responses may not have been related to the processing of “low-level” features such as lines. Moreover, these studies leave open the question of whether the early responses can be traced to V1 or to prestriate cortex, or both.

In our study, we tried to circumvent these problems by using stimuli constituted from straight lines, thus ensuring that the (early) “low-level” elements constituting the face or house stimuli were uniform throughout, differed in configuration alone and capable of stimulating strongly areas such as V1 and areas of prestriate visual cortex which have high concentrations of OS cells. We also tried to localize the source of the activity that we obtained, to establish whether both V1 and specialized areas of the prestriate cortex react with similar latencies. Our results show that, with both *Face* and *House* stimulation, not only is there a response from V1 but also from prestriate cortex, at around 40 ms after stimulus onset. This latency is similar to the earliest latency 28–32 ms in area V5 of prestriate visual cortex obtained after stimulation with fast motion (>22°s^−1^) (ffytche et al., 1995), 37 ms in V1 (Inui et al., 2006) and 27–44 ms in striate and prestriate cortex (Shgihara and Zeki, 2013), and it is even earlier than the ones reported by previous studies on face perception. Moreover, we have shown that there is a significant difference in the amplitude of the response to faces and houses, suggesting that the two stimuli are differentiated at this early time period. Our results therefore suggest that differentiation between face and house related activity in the visual brain is due to differences in amplitude of the response provoked by the two categories of stimuli rather than to differences in latency.

### Parallelism in the form pathway?

Our results thus make it plausible to suppose that the brain uses a parallel strategy, in addition to the hierarchical one, to process forms. Although they show that V1 and areas of the prestriate cortex give an early ERF component at around 40 ms, they do not show conclusively the operation of a parallel strategy since it is still conceivable that a signal from V1 originating at, say, 40 ms after stimulus onset would be processed hierarchically by striate and prestriate cortex within the 40–45 ms peak time window. But there are three lines of evidence which suggest strongly that the hierarchical strategy in constructing forms may be supplemented by a parallel one, in which the OS cells of V1 are not the sole source for the construction of forms in the specialized visual areas of the prestriate cortex.

#### MEG evidence

The first comes from the results of MEG experiments, which show that lines and more complex geometrical forms (rhombuses) constituted from them activate V1 and the visual areas of the prestriate cortex with similar latencies (Shgihara and Zeki, 2013). This is supplemented by fMRI evidence, which shows that lines, angles and rhombuses activate V1 and areas of the prestriate cortex with similar strengths, with angles producing the strongest and rhombuses the weakest activation in all (Shigihara and Zeki, 2014). This is contrary to what one might expect from the hierarchical doctrine, which would point to rhombuses as producing the strongest activation or to lines activating area V1 more strongly and rhombuses activating prestriate areas more strongly (Hubel and Wiesel, 1965). The results showing that V1 and areas V2 and V3 of prestriate cortex are engaged in parallel in processing geometric forms of increasing complexity (Shgihara and Zeki, 2013; Shigihara and Zeki, 2014) are consistent with the present results, which show that, likewise, V1 and specialized areas of prestriate cortex are also engaged in parallel in the processing of *Face* and *House* stimuli. One would conclude from this that there is no neat separation between the processing of “low-level” and “high-level” features and no parcellation of the former to V1 and the latter to “higher” areas in prestriate cortex.

#### Anatomical evidence

That there are parallel strategies within visual cortex, implied by the parallel anatomical connections from V1 and V2 (which are themselves interconnected) to say, V4 and V5, has long been acknowledged and its computational significance evaluated (e.g., Ballard et al., 1983; Grossberg, 1991). Much less attention has been given to the parallel inputs to V1 and areas of the prestriate visual cortex from the lateral geniculate nucleus (LGN) and the pulvinar, the latter of which may respond to visual (motion) stimuli before striate cortex does so (Ouellette and Casanova, 2006). This is surprising, since such pathways have been known to exist for a long time, from LGN (Cragg, 1969; Fries, 1981; Yukie and Iwai, 1981) and pulvinar (Cragg, 1969; Benevento and Rezak, 1976; Leventhal et al., 1980; Leh et al., 2008), both of which also receive input from the retina (Itaya and Van Hoesen, 1983; Nakagawa and Tanaka, 1984; Ouellette and Casanova, 2006; Baldwin et al., 2012). As well, the capacity of this “V1-bypassing” pathway to mediate a crude but conscious experience of at least visual motion, has been acknowledged (Barbur et al., 1993; Zeki and ffytche, 1998; Weiskrantz, 2004). The direct input to the motion sensitive area V5, from the LGN or the pulvinar (or both), leads in fact to a shorter latency activation of V5 (at between 28 and 32 ms) than does the input from the LGN to V1 (at about 75 ms), for fast moving stimuli (>22°s^−1^) (ffytche et al., 1995; Gaglianese et al., 2012), leading to the concept of *dynamic parallelism*. Hence, it becomes plausible to suppose that direct inputs from LGN and pulvinar to visual areas of the prestriate cortex with large concentrations of OS cells may deliver signals related to form vision directly to them (to areas such as V2, V3, and V3A) without passing through V1 (Schmid et al., 2009), just as they deliver fast motion-related signals directly to V5 (Beckers and Zeki, 1995; ffytche et al., 1995; Sincich et al., 2004). In light of our present results, they may also deliver signals that are critical for the perception of faces and houses directly to the relevant, specialized areas of the visual brain, especially since the pulvinar projections to the cortex are extensive and include the inferior temporal and the posterior parietal cortex, in addition to the occipital lobe (Leh et al., 2008). Here, it is interesting to note that the activation we observed, though it must remain tentative with respect to the precise subdivisions of the prestriate cortex because of the relatively poor spatial resolution of MEG, nevertheless suggests that V3, which contains high concentrations of OS cells (Zeki, 1978), and the areas which have been thought to play critical roles in face and house perception, were active at the early time windows. In such a scenario, the direct input to the latter areas, as well as the input through V1, would play different roles in processing stimuli but what role each input plays has yet to be determined.

#### Clinical evidence

The dominant role played by the classical visual pathway extending from the retina to V1 through the LGN undoubtedly also played an important role in emphasizing the hierarchical doctrine of form processing. This is especially so since lesions along this pathway, and particularly in V1, lead to blindnesses commensurate with the size and position of the lesions. Yet there is also evidence that lesions restricted to V2 and V3 lead to a comparable blindness (Horton and Hoyt, 1991), although such evidence is sparse because much more difficult to obtain, owing to the disposition of V2 and V3 in relation to V1, which means that damage to the latter usually also involves damage to the former. Hence V1 lesions do not have a monopoly in producing hemianopias.

### Asymmetric results with quadrant stimulation

It is well established that the right hemisphere is dominant for face perception (Kanwisher et al., 1997; McCarthy et al., 1997; Pitcher et al., 2007; Yovel et al., 2008). This is probably why sensor level analysis showed a larger RMS amplitude for *Face* than *House* in left quadrant presentation, since left quadrant presentations are mainly processed in the dominant (right) hemisphere for face perception. Right hemisphere dominance for face perception in the early response indicates that the cortical processing of face stimuli starts at the earliest stage of visual perception, around 40 ms post-stimulus.

### Problems arising from the present results

There are five possible problems worth mentioning here:

1. *Response time*: We note that the observed precedence in behavioral response times to *Faces* is not mirrored by an earlier cortical activation by *Faces* compared to *Houses*. However, behavioral response times may depend on later cortical activity rather than early ones (Johnson and Olshausen, 2003).
2. *Low-level feature differences in stimuli*: Although both *Face* and *House* stimuli consisted of the same elements, there are some unavoidable differences. *Face* stimuli had ears, which would activate a larger part of the field of view. By contrast, *House* stimuli had a window, which adds to the complexity of the inner small space; these differences might modulate the MEG amplitude differently (Tanskanen et al., 2005). Furthermore, each *Face* and *House* stimulus had only two versions and this limitation in the range of stimuli might have affected our results. However, these objections cannot account for the laterality which we observed: the difference in amplitude in the early components of the ERFs was significant only for stimuli presented in the left quadrant. The dominance of the right hemisphere for face perception can account for this laterality and leads us to discount the possibility that the responses which we have observed are due to low-level features. Moreover, Area V3, which contains OS cells and which has been linked to low-level feature selectivity, was not active with right quadrant presentation of face and house stimuli.
3. *N170 response*: N170 is the most prominent and well-known electromagnetic response for face perception. Although we detected the response (see, for example, Figure 4), we do not discuss it in this paper because it is not the main or even secondary aim of this work. The main finding here is that differences related to the processing of higher forms (faces and houses) were found to occur much earlier than N170 and were localizable to V1 and cortex outside it.
4. *Electrical activity in the retina*: Retinal activity can produce electromagnetic fields which can contaminate the MEG signals. The second wave (b-wave) produced by ON bipolar cells and the Muller cells could appear at around 25–50 ms (Crick and Khaw, 2003), which is the time window we are interested in. However, our responses were evidently not attributable to a retinal origin alone since the source was localized to occipital rather than frontal cortex.
5. *Source localization*: Estimated source locations at the peak time window for the four conditions are similar to each other (Figure 5 and Table 3), although there are differences in contour maps. There are two interpretations of this result: that the same brain areas were activated at the peak of the response but with different amplitudes or that the difference in source distributions was too small to be detected using our method (MSP). MSP uses source priors which are part of SPM-8. These priors restrict the variability of source distributions, a limitation which might have prevented us from detecting differences in source distribution between the four conditions in Figure 5. Although estimated sources at other time windows (Figures 6, 7) seem to include V3, OFA, FFA, and PPA, we have to exercise considerable caution in assigning the activity to specific cortical areas, given the limitations imposed by the use of MSP. We repeat, however, that our main aim was to learn whether sources could be localized to striate and prestriate cortex at the earliest time windows. The exact location of activity in prestriate cortex was of secondary interest, given that we had to sacrifice spatial resolution to obtain good temporal resolution.

### Unexpected results

There are two unexpected results. (1) In the source level analysis, sources were estimated in both left and right striate cortices (Figure 5). This result seems to conflict with the primary rule that striate cortex processes visual information originating from the contralateral visual field. There are two possible explanations for this. Firstly, the estimated source can be distributed to the opposite side of striate cortex, due to the limited spatial resolution of MEG and MSP source estimation (Cicmil et al., 2014). Secondly, our stimuli extended from 8.4 degrees on either side to the vertical meridian. A previous study has shown that there is a strip of central retina which projects to both hemispheres. The width of this strip is 1 to 6 degrees (Marzi et al., 2009), which may account for our results. (2) Sources in striate cortex for right hemifield presentation were not significant at *P*(FWE) < 0.05, although they were significant at *P*(unc.) < 0.001. This lack of significant activation (or weak activation) in striate cortex is surprising given that we do have significant prestriate activation. If anything, this result makes the case for parallel processing more emphatic for it suggests the possibility of a more potent input to prestriate than striate cortex with the face and house stimuli that we used. A parallel result may be found in the fact that signals from appropriate fast moving stimuli (>22°s^−1^) signal reach V5 directly and before they reach V1 (ffytche et al., 1995).

## Conclusion

Against this background, and in light of our earlier experiments (Shgihara and Zeki, 2013; Shigihara and Zeki, 2014), we suggest that a strong case can now be made for allocating to parallel strategies an important role in the cortical visual processing of forms as they relate to simple geometric forms as well as to higher level forms such as faces and houses.

Although we believe that our results suggest strongly that, in addition to the parallel connections between visual cortical areas, parallel anatomical inputs to V1 and to the specialized visual areas of the prestriate cortex from the LGN and the pulvinar, confers on the visual brain the capacity to process signals in parallel by V1 and areas of the prestriate cortex, we do not mean to imply that parallel processing in these different stations of the visual brain is also synchronous. There is good reason from psychophysical experiments (Moutoussis and Zeki, 1997; Arnold et al., 2001; Holcombe and Cavanagh, 2001; Viviani and Aymoz, 2001) to show that some attributes of the visual world are perceived before others, and hence that the “read-out” from the parallel processing systems may be asynchronous, in addition to being in parallel. Thus, parallel processing coupled to asynchronous “parallel read-outs,” introduces an interesting element into future hypotheses about the strategies used by the visual brain to construct an image of our world.

### Conflict of interest statement

The authors declare that the research was conducted in the absence of any commercial or financial relationships that could be construed as a potential conflict of interest.
