# Supplementary material for: Parallel processing of face and house stimuli by V1 and specialized visual areas: a magnetoencephalographic (MEG) study
Source: Front Hum Neurosci. 2014 Nov 7;8:901. doi: 10.3389/fnhum.2014.00901 (PMC4224090; doi:10.3389/fnhum.2014.00901)
Supplement: Supplementary file 1 [file DataSheet1.DOCX]

***Supplementary Material***

**Parallel processing of face and house stimuli by V1 and specialized visual areas: a magnetoencephalographic (MEG) study**

**Yoshihito Shigihara and Semir Zeki***

Wellcome Laboratory of Neurobiology, University College London, London WC1E 6BT

Authors: Yoshihito Shigihara and Semir Zeki

Affiliation: Wellcome Laboratory of Neurobiology, University College London, Gower Street, London, WC1E 6BT

* **Correspondence**: Semir Zeki; University College London, Gower Street, London, WC1E 6BT

s.zeki@ucl.ac.uk

**Supplementary Data**


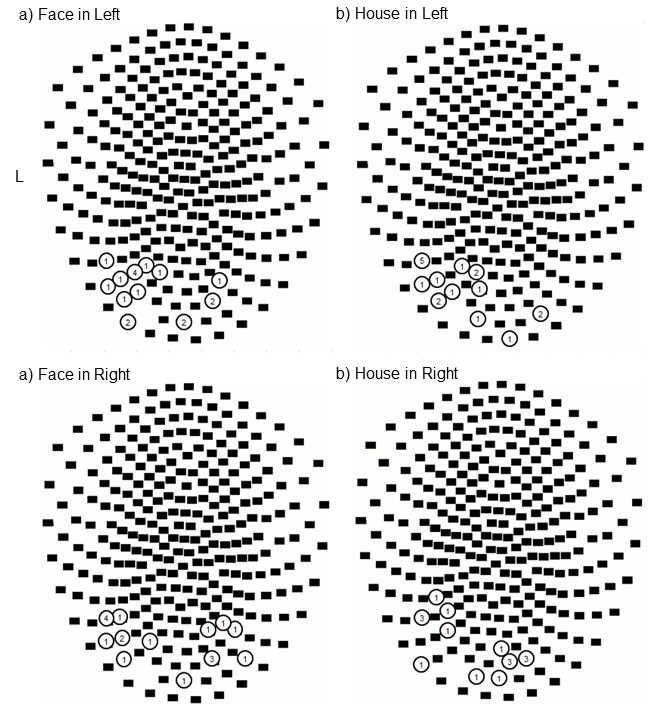


Figure S1. Sensor locations which detected the largest amplitude between 25 and 50 ms post stimulus (SOI). Circled numbers indicate the number of subjects who showed the largest amplitude at that sensor. This sensor location does not indicate precisely the location of the activated brain areas because the sensors are axial type gradiometers. L, left.

**
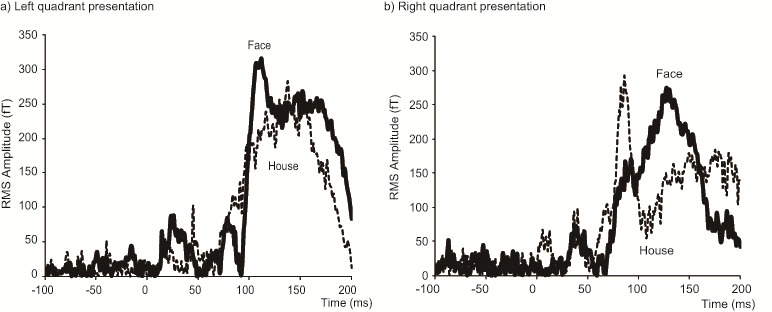
**

Figure S2. Representative example of averaged RMS amplitude from a single subject (Subject #12). Each condition shows a peak before 50 ms after stimulus onset. Time courses of averaged RMS amplitude across 18 subjects is shown in Figure 4.

Table S1. Results of ANOVA, post hoc t-test, paired t-test between two forms (Face vs House), and Peak time of ERF.

|  |  |  |  | ANOVA | | |  |  | Post hoc t-test | | | | | | | | | | |  | Peak |
| --- | --- | --- | --- | --- | --- | --- | --- | --- | --- | --- | --- | --- | --- | --- | --- | --- | --- | --- | --- | --- | --- |
|  |  |  |  |  |  |  |  |  | Time window (ms) | | | | | | | | | | |  |  |
| Comparison | Stimulation | |  | df | F | p |  |  | Baseline | 0-5 | 5-10 | 10-15 | 15-20 | 20-25 | 25-30 | 30-35 | 35-40 | 40-45 | 45-50 |  | (ms) |
| Within form | Left | Face |  | 10 | 5.377 | <0.001 |  | t |  | 0.176 | 0.989 | 0.881 | 0.752 | 2.227 | 3.192 | 3.505 | 3.829 | 4.683 | 3.289 |  | 37.2 |
|  |  |  |  |  |  |  |  | p |  | 0.861 | 0.324 | 0.380 | 0.453 | 0.027* | 0.002* | 0.001* | <0.001* | <0.001* | 0.001* |  |  |
|  |  | House |  | 10 | 3.682 | <0.001 |  | t |  | 0.006 | 0.298 | 0.745 | 1.664 | 1.445 | 1.275 | 2.237 | 4.090 | 3.173 | 1.509 |  | 39.7 |
|  |  |  |  |  |  |  |  | p |  | 0.995 | 0.766 | 0.457 | 0.098 | 0.150 | 0.204 | 0.027* | <0.001* | 0.002* | 0.133 |  |  |
|  | Right | Face |  | 10 | 6.403 | <0.001 |  | t |  | 0.115 | 0.562 | 0.961 | 1.781 | 2.988 | 3.047 | 3.263 | 3.407 | 3.588 | 4.925 |  | 47.2 |
|  |  |  |  |  |  |  |  | p |  | 0.909 | 0.575 | 0.338 | 0.077 | 0.003* | 0.003* | 0.001* | 0.001* | <0.001* | <0.001* |  |  |
|  |  | House |  | 10 | 6.403 | <0.001 |  | t |  | 0.575 | 1.463 | 1.056 | 0.076 | 0.515 | 1.103 | 2.478 | 3.508 | 4.716 | 4.814 |  | 44.7 |
|  |  |  |  |  |  |  |  | p |  | 0.566 | 0.145 | 0.292 | 0.940 | 0.607 | 0.272 | 0.014* | 0.001* | <0.001* | <0.001* |  |  |
| Between forms | Left | Face vs House |  |  |  |  |  | t | 0.222 | 0.409 | 1.696 | 0.585 | -0.608 | 1.219 | 2.329 | 1.566 | 0.484 | 1.873 | 1.571 |  |  |
|  |  |  |  |  |  |  |  | p | 0.827 | 0.688 | 0.108 | 0.566 | 0.551 | 0.240 | 0.032* | 0.136 | 0.635 | 0.078 | 0.135 |  |  |
|  | Right | Face vs House |  |  |  |  |  | t | 0.746 | 0.866 | 0.847 | 0.165 | 1.039 | 1.464 | 1.190 | 0.502 | 0.027 | 0.554 | 0.221 |  |  |
|  |  |  |  |  |  |  |  | p | 0.466 | 0.399 | 0.409 | 0.871 | 0.313 | 0.162 | 0.250 | 0.622 | 0.979 | 0.587 | 0.828 |  |  |

Ryan correction was applied for post hoc t-tests. An asterisk indicates significant difference (p < 0.05).

Table S2. Sources locations which were responsible for producing the early components of ERFs

|  |  | Time |  |  | Cluster |  |  |  | Peak |  |  |  | Coordinate |  |  | Brain |
| --- | --- | --- | --- | --- | --- | --- | --- | --- | --- | --- | --- | --- | --- | --- | --- | --- |
| Stimulation |  | window (ms) |  | p(FWE) | kE | p(unc) |  | p(FWE) | T | p(unc) |  | X | Y | Z |  | Reigion |
| Left | Face | 35-40 |  | 0.015 | 376 | 0.026 |  | **0.008** | 6.27 | < 0.001 |  | 36 | -80 | 6 |  | Prestriate |
|  |  |  |  | 0.070 | 159 | 0.128 |  | **0.030** | 5.42 | < 0.001 |  | -40 | -82 | -12 |  | Prestriate |
|  |  |  |  | 0.005 | 555 | 0.009 |  | **0.038** | 5.28 | < 0.001 |  | 6 | -80 | 14 |  | Striate |
|  |  |  |  |  |  |  |  | **0.040** | 5.24 | < 0.001 |  | -6 | -78 | 18 |  | Striate |
|  |  |  |  |  |  |  |  | 0.103 | 4.65 | < 0.001 |  | 4 | -76 | 24 |  | Prestriate |
|  |  |  |  | 0.047 | 210 | 0.084 |  | **0.040** | 5.25 | < 0.001 |  | 26 | -94 | -18 |  | Prestriate |
|  |  |  |  |  |  |  |  | 0.300 | 3.93 | 0.001 |  | 14 | -82 | -14 |  | Prestriate |
|  |  |  |  | 0.268 | 25 | 0.550 |  | 0.092 | 4.72 | 0.000 |  | -12 | -86 | -8 |  | Prestriate |
|  |  |  |  | 0.370 | 5 | 0.815 |  | 0.359 | 3.80 | 0.001 |  | -40 | -56 | -12 |  | Prestriate |
|  |  |  |  | 0.300 | 17 | 0.630 |  | 0.393 | 3.73 | 0.001 |  | -20 | -80 | -8 |  | Prestriate |
|  | House | 35-40 |  | 0.088 | 158 | 0.249 |  | **0.005** | 6.29 | < 0.001 |  | -40 | -74 | -10 |  | Prestriate |
|  |  |  |  |  |  |  |  | 0.188 | 4.00 | < 0.001 |  | -46 | -60 | -12 |  | Prestriate |
|  |  |  |  | 0.211 | 29 | 0.638 |  | **0.020** | 5.40 | < 0.001 |  | -38 | -50 | -14 |  | Prestriate |
|  |  |  |  | 0.036 | 339 | 0.099 |  | **0.039** | 4.99 | < 0.001 |  | 36 | -80 | 6 |  | Prestriate |
|  |  |  |  |  |  |  |  | 0.056 | 4.77 | < 0.001 |  | 36 | -80 | -4 |  | Prestriate |
|  |  |  |  | 0.026 | 418 | 0.070 |  | **0.040** | 4.98 | < 0.001 |  | 6 | -80 | 14 |  | Striate |
|  |  |  |  |  |  |  |  | 0.053 | 4.81 | < 0.001 |  | -6 | -78 | 16 |  | Striate |
|  |  |  |  |  |  |  |  | 0.062 | 4.70 | < 0.001 |  | -2 | -74 | 8 |  | Striate |
|  |  |  |  | 0.065 | 215 | 0.182 |  | 0.053 | 4.81 | < 0.001 |  | 26 | -94 | -18 |  | Striate |
|  |  |  |  |  |  |  |  | 0.074 | 4.60 | < 0.001 |  | 20 | -88 | -12 |  | Prestriate |
|  |  |  |  |  |  |  |  | 0.093 | 4.46 | < 0.001 |  | 12 | -82 | -14 |  | Prestriate |
|  |  |  |  | 0.254 | 11 | 0.790 |  | 0.090 | 4.48 | < 0.001 |  | 34 | -62 | -16 |  | Prestriate |
|  |  |  |  | 0.271 | 6 | 0.855 |  | 0.134 | 4.23 | < 0.001 |  | -12 | -86 | -8 |  | Prestriate |
|  |  |  |  | 0.203 | 33 | 0.613 |  | 0.137 | 4.21 | < 0.001 |  | -20 | -78 | -8 |  | Prestriate |
|  |  |  |  | 0.260 | 9 | 0.814 |  | 0.148 | 4.16 | < 0.001 |  | 48 | -56 | -10 |  | Prestriate |
|  |  |  |  | 0.298 | 1 | 0.954 |  | 0.181 | 4.03 | < 0.001 |  | -36 | -62 | -16 |  | Prestriate |
|  |  |  |  | 0.291 | 2 | 0.927 |  | 0.204 | 3.94 | 0.001 |  | 38 | -66 | -20 |  | Prestriate |
|  |  |  |  | 0.285 | 3 | 0.906 |  | 0.265 | 3.76 | 0.001 |  | -10 | -82 | -12 |  | Prestriate |
|  |  |  |  | 0.298 | 1 | 0.954 |  | 0.295 | 3.68 | 0.001 |  | -2 | -78 | 26 |  | Prestriate |
| Right | Face | 45-50 |  | 0.127 | 94 | 0.263 |  | **0.011** | 5.98 | < 0.001 |  | -40 | -82 | -12 |  | Prestriate |
|  |  |  |  | 0.323 | 9 | 0.756 |  | **0.049** | 5.06 | < 0.001 |  | 34 | -62 | -16 |  | Prestriate |
|  |  |  |  | 0.057 | 195 | 0.114 |  | 0.087 | 4.71 | < 0.001 |  | 36 | -80 | -4 |  | Prestriate |
|  |  |  |  |  |  |  |  | 0.098 | 4.63 | < 0.001 |  | 36 | -80 | 6 |  | Prestriate |
|  |  |  |  | 0.239 | 31 | 0.528 |  | 0.143 | 4.39 | < 0.001 |  | -6 | -86 | 18 |  | Striate |
|  |  |  |  | 0.233 | 33 | 0.514 |  | 0.158 | 4.32 | < 0.001 |  | 6 | -84 | 20 |  | Prestriate |
|  |  |  |  | 0.271 | 21 | 0.611 |  | 0.209 | 4.13 | < 0.001 |  | -20 | -80 | -8 |  | Prestriate |
|  |  |  |  | 0.384 | 1 | 0.937 |  | 0.294 | 3.89 | 0.001 |  | 38 | -66 | -20 |  | Prestriate |
|  |  |  |  | 0.373 | 2 | 0.903 |  | 0.298 | 3.88 | 0.001 |  | -12 | -86 | -8 |  | Striate |
|  | House | 40-45 |  | 0.128 | 89 | 0.225 |  | **0.003** | 6.87 | < 0.001 |  | -40 | -82 | -12 |  | Striate |
|  |  |  |  | 0.305 | 18 | 0.598 |  | 0.074 | 4.91 | < 0.001 |  | -20 | -80 | -8 |  | Prestriate |
|  |  |  |  | 0.025 | 279 | 0.041 |  | 0.096 | 4.74 | < 0.001 |  | -6 | -86 | 18 |  | Striate |
|  |  |  |  |  |  |  |  | 0.101 | 4.71 | < 0.001 |  | 6 | -84 | 20 |  | Striate |
|  |  |  |  |  |  |  |  | 0.157 | 4.42 | < 0.001 |  | -2 | -74 | 8 |  | Striate |
|  |  |  |  | 0.083 | 133 | 0.142 |  | 0.202 | 4.25 | < 0.001 |  | 24 | -90 | -14 |  | Prestriate |
|  |  |  |  |  |  |  |  | 0.342 | 3.88 | 0.001 |  | 16 | -88 | -6 |  | Striate |
|  |  |  |  |  |  |  |  | 0.353 | 3.85 | 0.001 |  | 14 | -82 | -14 |  | Prestriate |
|  |  |  |  | 0.379 | 6 | 0.781 |  | 0.241 | 4.13 | < 0.001 |  | -38 | -50 | -14 |  | Prestriate |
|  |  |  |  | 0.363 | 8 | 0.741 |  | 0.298 | 3.98 | < 0.001 |  | 34 | -82 | 12 |  | Prestriate |

Statistical values, Cluster size, Location, MNI co-ordinates are displayed. In this table, all sources are peak level significant at p < 0.001 (uncorrected). Bold numbers show the peak level significant at p < 0.05 (FWE corrected).
